# 肺岩宁对人高转移95-D肺癌细胞上皮-间质细胞标志因子表达的影响

**DOI:** 10.3779/j.issn.1009-3419.2011.06.01

**Published:** 2011-06-20

**Authors:** 晓珍 赵, 中华 吴, 振晔 徐, 中奇 王, 继 吴, 婉 苏

**Affiliations:** 1 200032 上海，上海中医药大学附属龙华医院肿瘤科，上海中医药研究院肿瘤研究所 Department of Oncology Section of Longhua Hospital, Cancer Institute of Chinese Medicine, Shanghai 200032, China; 2 201203 上海，上海中医药大学科技实验中心 Science and Technology Experiment Center, Shanghai University of Traditional Chinese Medicine, Shanghai 201203, China

**Keywords:** 肺肿瘤, 中医, 上皮-间质细胞转化, Alpha-catenin、beta-catenin、E-Cadherin、N-cadherin、Fibronectin、Vimentin, 肿瘤转移, Lung neoplasms, Epithelial-mesenchymal transition, Alpha-catenin; beta-catenin; E-Cadherin; N-cadherin; Fibronectin; Vimentin, Tumor metastasis

## Abstract

**背景与目的:**

前期体内实验证实肺岩宁方具有上调上皮细胞标志因子、Alpha-catenin、beta-catenin、E-Cadherin和下调间质细胞标志因子N-cadherin、Fibronectin的作用，而对Vimentin无明显作用。在此基础上，本研究旨在进一步以人高转移95-D肺癌细胞为研究对象，探讨肺岩宁方对上皮-间质细胞标志因子基因和蛋白表达的影响。

**方法:**

采用不同浓度肺岩宁血清干预治疗人高转移肺癌细胞95-D的基础上，采用Real-time PCR及Western blot方法检测对上皮-间质细胞标志因子Alpha-catenin、beta-catenin、E-Cadherin、N-cadherin、Fibronectin、Vimentin基因和蛋白表达的影响。

**结果:**

Real-time PCR结果表明：与对照组相比较，20%肺岩宁处理组Alpha-catenin表达上调（*P* < 0.05），20%、25%肺岩宁处理组E-Cadherin表达上调（*P* < 0.05, *P* < 0.01）；各肺岩宁处理组beta-catenin表达无明显改变；5%、10%肺岩宁处理组间质细胞标志因子Vimentin表达下调（*P* < 0.01），各肺岩宁处理组N-cadherin、Fibronectin无明显改变；Western blot结果表明：5%、10%、15%、20%、25%肺岩宁处理组E-Cadherin蛋白表达上调（*P* < 0.01），各肺岩宁处理组Alpha-catenin、beta-catenin蛋白表达无明显改变；5%、10%肺岩宁处理组N-cadherin、Fibronectin蛋白表达下调（*P* < 0.01），各肺岩宁处理组Vimentin蛋白表达无明显改变。

**结论:**

肺岩宁方通过调控部分上皮-间质细胞标志因子的表达，从而抑制肿瘤异质粘附能力和运动能力来参与肺癌的侵袭和转移。

## Abstract (en)

**Background and objective:**

Our previous *in vivo* experiment (paper has been published) has confirmed Feiyanning's regulation effect on epithelial cell markers Alpha-catenin, beta-catenin, E-Cadherin and mesenchymal cell markers N-cadherin, Fibronectin, vimentin. Based on previous study, the objective of this study is to further investigate the expression of epithelial-mesenchymal cell marker gene and protein intervened by Feiyanning in highly metastatic lung cancer cells 95-D *in vitro*.

**Methods:**

Human highly metastatic lung cancer 95-D cells were treated with different concentrations of Feiyanning *in vitro*, and then Real-time PCR and Western blot methods were used to detect mRNA and protein expression of epithelial-mesenchymal cell marker factor Alpha-catenin, beta-catenin, E-Cadherin, N-cadherin, Fibronectin and vimentin.

**Results:**

Real-time PCR results showed that compared with the control group, Feiyanning at 20% concentration remarkably up-regulated the expression of Alpha-catenin (*P* < 0.05), Feiyanning at 20% and 25% concentrations remarkably up-regulated the expression of E-Cadherin (*P* < 0.05, *P* < 0.01), Feiyanning had no significantly effect on beta-catenin at any concentration, 5% and 10% Feiyanning at 5% and 10% concentrations significantly dow-regulated the expression of mesenchymal cell marker vimentin factor (*P* < 0.01), Feiyanning had no regulatory role on N-cadherin and Fibronectin at any concentration. Western blot results showed that Feiyanning at 5%, 10%, 15%, 20% and 25% concentrations significantly increased E-Cadherin protein expression (*P* < 0.01), but had no regulation effect on Alpha-catenin and beta-catenin protein expression; Feiyanning at 5% and 10% concentrations had a downregulation effect on N-cadherin and Fibronectin protein expression (*P* < 0.01), and had no regulation effect on vimentin protein expression.

**Conclusion:**

Feiyanning play a role in inhibiting the adhesion of tumor heterogeneity and the athletic ability by regulating epithelial-mesenchymal cell marker factor expression, and thus inhibit the invasion and metastasis of lung cancer.

肿瘤转移的机理一直是肿瘤研究领域的热点，而如何阻止与预防癌细胞的转移更是研究者关注的焦点。上皮-间质细胞转化（epithelial-mesenchymal transition, EMT）是指上皮组织被各种刺激诱发的上皮细胞向间质细胞表型的转化。在此过程中上皮细胞失去极性及细胞-细胞、细胞-基质间的粘附性，转化为间质细胞，获得迁移与侵袭能力；而在癌化过程中，癌细胞通过具有锌指结构的Snail、Slug、Sip-1和具有螺旋-环状-螺旋结构的Twist与E-Cadherin启动子部分的E-boxes相结合，导致如上皮细胞标志因子E-Cadherin基因的失活和表达的下调，同时伴随间质细胞标志因子Cadherin分子的转化如N-Cadherin等。因此，EMT如果发生了就具备了转移的特性，所以研究^[1, 2]^认为EMT是导致癌细胞发生转移的导火线。

肺岩宁方是徐振晔教授领导科研课题组在三十余年的临床实践中总结出以益气养精、解毒抗癌为治法，防治肺癌转移的中药复方，在临床上取得良好的抗肿瘤转移的疗效，使患者的生存期得到有效延长，生存质量明显得到改善^[3-5]^。

本课题组在以往的研究中成功构建C57 Lewis肺转移模型，实验^[6, 7]^结果提示肺岩宁方具有明显上调上皮-间质细胞转化过程中上皮细胞标志因子α-catenin、β-catenin、E-Cadherin和下调间质细胞标志因子N-cadherin、Fibronectin的作用，说明在肺癌转移的过程中伴随着上皮-间质细胞表型的转化，而肺岩宁方在一定程度上可以调控EMT过程的发生。本研究将以人高转移肺癌细胞95-D作为研究对象，进一步研究肺岩宁方调控EMT的机制。

## 1 材料与方法

### 1.1 细胞系及主要试剂

人高转移95D肺癌细胞，均由上海中科院细胞所提供。RPMI1640（GIBICO公司），新鲜小牛血清（杭州四季青公司），胰蛋白酶（华美生物制品公司），Trizol（Invitrogen公司），逆转录试剂（MBI公司），TaqDNA聚合酶及SYBR Green I荧光定量PCR试剂（大连宝生物工程公司），鼠抗人α-catenin抗体、鼠抗人β-catenin抗体、鼠抗人E-Cadherin抗体、鼠抗人Vimentin抗体、鼠抗人Fibronectin抗体、鼠抗人N-cad-herin抗体均购买于Santa cruz。

### 1.2 药物

肺岩宁方：由生黄芪、女贞子、蜂房、山慈菇、七叶一枝花、黄精等药物组成。龙华医院中药房提供；顺铂（DDP）：20 mg/瓶，齐鲁制药厂产品，批号：0501003。

### 1.3 细胞培养

将人高转移95D细胞培养于含有10%胎牛血清的RPMI-1640培养液中（pH7.2-7.4），在37 ℃、5%CO_2_和饱和湿度条件下，细胞呈贴壁生长，待其铺满瓶时以0.25%胰蛋白酶消化传代，取对数生长期的细胞进行实验。

### 1.4 肺岩宁方含药血清的制备

雄性SD大鼠10只，随机分为对照组和肺岩宁方组，每组5只，组间体重差异无统计学意义，肺岩宁方组按体重灌胃给予肺岩宁方药液（相对于人临床用药剂量的8倍），对照组给予等体积的生理盐水灌胃，2次/d，共3 d，最后一次给药前禁食12 h（不禁水）。末次给药后1 h，腹腔注射2%戊巴比妥钠5 mL麻醉，无菌条件下腹主动脉取血，立即注入无菌试管中，室温静置，待凝血坚固，血清析出后，吸出上清离心处理（3, 000 r/min，10 min，离心半径为10 cm），取上清3 mL左右，同组血清混合，置于无菌试管中，56 ℃水浴中灭活30 min，用0.22 μm微孔滤膜除菌，分装，密封，-20 ℃冰箱冻存备用。

### 1.5 实验分组

将细胞密度调至1×10^6^个/mL后均匀接种于96孔培养板中，6个复孔，置入37 ℃、5%CO_2_含10%新生小牛血清的RPMI-1640培养液中培养48 h，待细胞生长旺盛时，离心去掉上清液，分别加入各组不同浓度的细胞培养液，根据预实验依据含药血清浓度由低到高分为5组，使其终浓度为体积分数5%、10%、15%、20%、25%，另设对照组DDP化疗阳性对照组（2 μg/mL）和15%空白血清对照组，于培养箱中继续培养48 h后收集细胞检测。

### 1.6 Real-time

PCR检测各组细胞α-catenin、β-catenin、E-Cadherin、N-cadherin、Fibronectin、Vimentin RNA表达按照说明用Trizol等试剂从细胞中提取总RNA，以75%乙醇洗涤后再用DEPC处理水溶解RNA的制备：取各组的对数生长期细胞1×10^7^个，加入1 mL Trizol匀浆后按照说明书提取总RNA，1 μL总RNA在20 μL体系中按照标准程序进行逆转录，逆转录酶采用M-MuLV。各基因mRNA拷贝数检测采用实时定量PCR，反应体积为20 μL，其中含终浓度为200 nmol/L的上下游引物和2×的荧光标记物SYBRGreen PCR Master Mix，扩增反应用SYBR Green I定量PCR试剂盒，各反应管放入ABI-7300定量PCR仪。内参选用GAPDH引物由上海Invitrogen公司设计合成。引物序列见表 1。

**1:** 各基因PCR引物序列 Primer sequence of different gene

Gene	Primer sequence (5'->3')
*GAPDH*	Sense: ATGGGGAAGGTGAAGGTCG Antisense: GGGGTCATTGATGGCAACAATA
*α-catenin*	Sense: CCATGCAGGCAACATAAACTTC Antisense: GGCTCCAACAGTCTCTCAACT
*β-catenin*	Sense: AGGGATTTTCTCAGTCCTTC Antisense: CATGCCCTCATCTAATGTCT
*E-cadherin*	Sense: CCCACCACGTACAAGGGTC Antisense: CTGGGGTATTGGGGGCATC
*N-cadherin*	Sense: GCCCACGGTAACAACCTCTT Antisense: GAGGAGTCAGTGAAGGAGTCA
*Fibronectin*	Sense: GAAGCTCTCTCTCAGACAACCA Antisense: GCCCACGGTAACAACCTCTT
*Vimentin*	Sense: GAACGCCAGATGCGTGAAATG Antisense: CCAGAGGGAGTGAATCCAGATTA

### 1.7 Western blot方法检测各组细胞α-catenin、β-catenin、E-Cadherin、N-cadherin、Fibronectin、Vimentin蛋白表达

提取适量细胞中加入预冷的含抑制剂的蛋白质抽提试剂，轻轻摇动5 min。转移细胞悬浮液到离心管中，冰浴下摇动15 min进行裂解。上清液采用BCA蛋白定量，取蛋白进行SDS-PAGE电泳胶分离后转移到PVDF膜，膜封闭后加入多克隆抗体4 ℃过夜，然后加入相应二抗后膜在将膜置于反应液中室温孵育5 min。去除过量的溶液，将膜夹在两塑料薄膜之间，以X光胶片曝光。图片扫描保存为电脑文件，并用分析软件将图片上每个特异条带灰度值的数字化。

### 1.8 数据处理统计分析

基因表达采用2^-△△ct^法对实验样品进行相对定量。实验数据以Mean±SD表示，根据数据类型选择重复测量设计方差分析或单因素方差分析的统计方法，采用SPSS 14.0统计软件进行统计处理。以*P* < 0.05为具有统计学差异。

## 2 结果

### 2.1 肺岩宁处理后人肺高转移95-D细胞上皮细胞标志因子α-catenin、β-catenin、E-Cadherin基因表达的影响

与对照组相比，20%肺岩宁处理组α-catenin表达上调（*P* < 0.05），20%、25%肺岩宁处理组E-Cadherin表达上调（*P* < 0.05, *P* < 0.01），而各肺岩宁处理组β-catenin的表达无明显差异，阳性对照DDP组β-catenin表达上调（*P* < 0.01）（图 1）。

**1:**
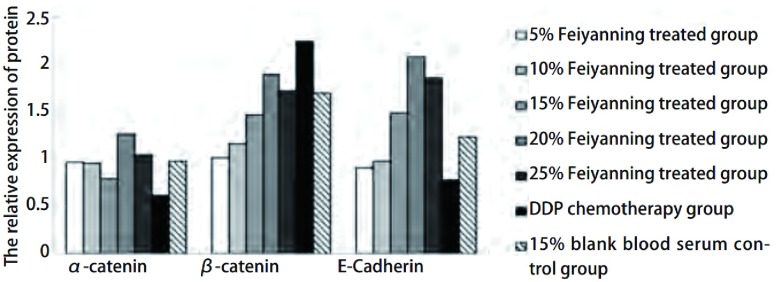
不同组别上皮细胞标志因子*α*-catenin、*β*-catenin、E-Cadherin基因表达变化。直方图显示20%肺岩宁处理组*α*-catenin表达增加，20%、25%肺岩宁处理组E-Cadherin表达增加，DDP处理组*β*-catenin表达增加。 The gene expression of epithelial cell markers *α*-catenin, *β*-catenin, E-Cadherin in different groups. The relative experssion of *α*-catenin mRNA was increased in 20% Feiyanning treated group; E-Cadherin mRNA were increased in 20% and 25% Feiyanning treated groups; *β*-catenin mRNA was increased in DDP chemotherapy group.

### 2.2 肺岩宁处理后人肺高转移95-D细胞上皮细胞标志因子α-catenin、β-catenin、E-Cadherin蛋白表达的影响

与对照组相比，5%、10%、15%、20%肺岩宁处理组E-Cad-herin表达上调（*P* < 0.01），而各肺岩宁处理组α-catenin、β-catenin的表达无明显差异（图 2）。

**2:**
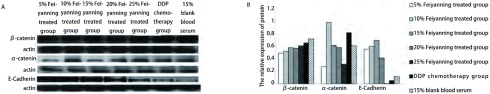
不同组别上皮细胞标志因子*β*-catenin、*α*-catenin、E-Cadherin蛋白表达变化。A：以actin为内参照，上皮细胞标志因子蛋白电泳图；B：直方图显示5%、10%、15%、20%肺岩宁处理组E-Cadherin蛋白表达增加，各肺岩宁处理组中*α*-catenin、*β*-catenin表达无差别。 The relative expression of *β*-catenin, *α*-catenin, E-Cadherin protein in different groups. A: actin as an internal reference, the figure of pro-tein electrophoresis to the epithelial cell marker factor; B: The relative expressionof E-Cadherin protein were increased in 5%, 10%, 15%, 20% Fei-yanning treated groups, and there was no difference for *α*-catenin, *β*-catenin in Feiyanning treated groups.

### 2.3 肺岩宁处理后人肺高转移95-D细胞间质细胞标志因子N-cadherin、Fibronectin、Vimentin基因表达的影响

与对照组相比，5%、10%肺岩宁处理组Vimentin表达下调（*P* < 0.01），而各肺岩宁处理组N-cadherin、Fibronectin的表达无明显差异（图 3）。

**3:**
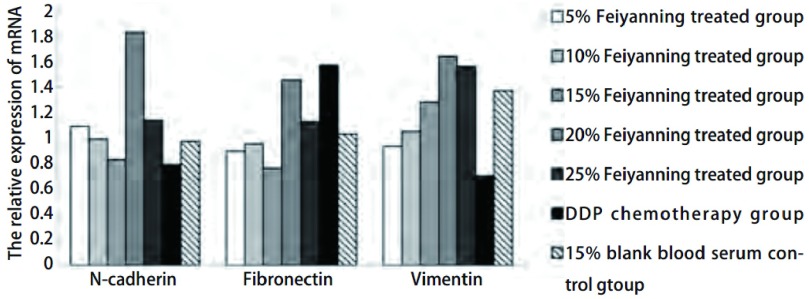
不同组别间质细胞标志因子N-cadherin、Fibronectin、Vimentin基因表达变化。直方图显示5%、10%肺岩宁处理组Vimentin表达降低，各肺岩宁处理组中N-cadherin、Fibronectin表达无明显变化。 The gene expression of mesenchymal cell markers of N-cadherin, Fibronectin, Vimentin in different groups. The relative experssion of Vimentin mRNA were decreased in 5% and 10% Feiyanning treated groups, but there was no difference for N-cadherin and Fibronectin in Feiyanning treated groups.

### 2.4 肺岩宁处理后人肺高转移95-D细胞间质细胞标志因子N-cadherin、Fibronectin、Vimentin蛋白表达的影响

与对照组相比，5%、10%肺岩宁处理组N-cadherin、Fibro-nectin表达下调（*P* < 0.01），而各肺岩宁处理组Vimentin的表达无明显差异（图 4）。

**4:**
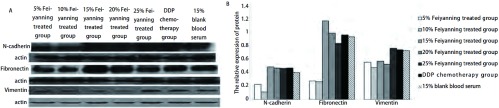
不同组别间质细胞标志因子N-cadherin、Fibronectin、蛋白表达变化。A：以actin为内参照，间质细胞标志因子蛋白电泳图；B：直方图显示5%、10%肺岩宁处理组N-cadherin、Fibronectin蛋白表达下降，各肺岩宁处理组中Vimentin表达无明显变化。 The relative expression of N-cadherin, Fibronectin, Vimentin protein in different groups. A: actin as an internal reference, the figure of protein electrophoresis to the mesenchymal cell markers factor; B: The relative expression of N-cadherin and Fibronectin protein were deceased in 5% and 10% Feiyanning treated groups, but there was no difference for Vimentin in Feiyanning treated groups.

## 3 讨论

医学研究^[8]^表明，肿瘤的转移是导致死亡和手术失败的主要原因。所以明确肿瘤转移的机理及进一步研究抗肿瘤转移的药物成为癌症研究领域的热点。中医药抗肿瘤的研究主要方向之一即在于中药方剂对肿瘤转移的抑制上，我们课题组三十余年临床探索认为精气两亏是导致肿瘤转移的重要因素之一，采用益气养精为主要治则的肺岩宁方即是在此理论指导下产生并被实践证明有效的方剂。

EMT是指从具有极性的上皮细胞转换成具有移行能力的间质细胞的一个过程。研究^[9, 10]^发现其不仅参与了胚胎的发育、组织的形成，而且参与了肿瘤细胞转移的发生。

EMT发生过程中的重要性标志就是E-Cadherin表达的减少和缺失。E-Cadherin是钙粘蛋白家族的一个成员，它在维持上皮细胞的极性及增强细胞间的粘附中具有重要作用，它表达的减少可以导致细胞极性的消失和细胞粘附能力的下降，在多项研究^[10-16]^中已经证实E-Cadherin的表达与肿瘤的侵袭能力成负相关，上皮细胞极性消失的同时伴随着间质细胞标志因子Vimentin、N-cadherin、Fibronectin等表达的增加，从而促使EMT过程的发生，导致肿瘤转移的开始。

本实验结果表明：以人肺癌高转移95-D细胞为研究对象，和正常血清对照组相比，肺岩宁在mRNA和蛋白质水平具有上调上皮细胞标志因子E-Cadherin的作用；在mRNA水平具有下调间质细胞标志因子Vimentin的作用；在蛋白质水平具有下调N-cadherin、Fibronectin的作用，而对上皮细胞标志因子α-catenin、β-catenin却无调控作用。由此我们认为：①人高转移肺癌95-D细胞转移过程的发生中伴随着EMT标志因子表达的改变；②中药复方肺岩宁通过上调E-Cadherin、下调Vimentin、N-cadherin、Fibronectin的表达而达到抑制肿瘤转移的作用；③mRNA水平和蛋白质水平肺岩宁调控的标志因子存在差异；④肺岩宁方调控EMT过程的发生是多靶点的、多层次的。

因此，我们将在今后的研究中一方面围绕调控EMT的信号途径阐释复方肺岩宁方抗肿瘤转移的机理，另一方面以Vimentin为例，深入探讨肺岩宁方在转录和蛋白质水平调控机制存在差异的原因。进一步揭示中医药抗肿瘤转移的机理。
